# Fulgor: a fast and compact *k*-mer index for large-scale matching and color queries

**DOI:** 10.1186/s13015-024-00251-9

**Published:** 2024-01-22

**Authors:** Jason Fan, Jamshed Khan, Noor Pratap Singh, Giulio Ermanno Pibiri, Rob Patro

**Affiliations:** 1 Department of Computer Science, University of Maryland, College Park, MD 20742 USA; 2 DAIS, Ca’ Foscari University of Venice, Venice, Italy; 3 ISTI-CNR, Pisa, Italy

**Keywords:** *k*-mers, Colored compacted *de Bruijn* graph, Compression, Read-mapping

## Abstract

The problem of sequence identification or matching—determining the subset of reference sequences from a given collection that are likely to contain a short, queried nucleotide sequence—is relevant for many important tasks in Computational Biology, such as metagenomics and pangenome analysis. Due to the complex nature of such analyses and the large scale of the reference collections a resource-efficient solution to this problem is of utmost importance. This poses the threefold challenge of representing the reference collection with a data structure that is efficient to query, has light memory usage, and scales well to large collections. To solve this problem, we describe an efficient *colored de Bruijn* graph index, arising as the combination of a *k*-mer dictionary with a compressed inverted index. The proposed index takes full advantage of the fact that unitigs in the colored compacted de Bruijn graph are *monochromatic* (i.e., all *k*-mers in a unitig have the same set of references of origin, or *color*). Specifically, the unitigs are kept in the dictionary in color order, thereby allowing for the encoding of the map from *k*-mers to their colors in as little as 1 + *o*(1) bits per unitig. Hence, one color per unitig is stored in the index with almost no space/time overhead. By combining this property with simple but effective compression methods for integer lists, the index achieves very small space. We implement these methods in a tool called Fulgor, and conduct an extensive experimental analysis to demonstrate the improvement of our tool over previous solutions. For example, compared to Themisto—the strongest competitor in terms of index space vs. query time trade-off—Fulgor requires significantly less space (up to 43% less space for a collection of 150,000 *Salmonella enterica* genomes), is at least twice as fast for color queries, and is 2–6$\times$ faster to construct.

## Introduction

At the core of many metagenomic and pan-genomic analyses is *read-mapping*, the atomic operation that assigns observed sequence reads to putative genome(s) of origin. A wide range of methods have been developed for mapping reads to large collections of reference genomes. Of note, alignment-based methods, though accurate [1, 2], are relatively computationally intensive as they must provide the ability to *locate* the read on each genome and compute an approximate match. A queried read must, in fact, be matched with low edit-distance against a sub-string of some reference genome in the collection. For alignment, the index is also required to report the position of this match. As a matter of fact, there are no practical aligners in the literature that scale to large genomic collections efficiently.

Fortunately, *alignment-free* techniques have become popular and widespread for metagenomic analyses [3–8]. These methods generally work by avoiding alignment altogether, and replacing it with strategies for matching (exactly or approximately) substrings, signatures, or sketches between the queries and the referenced sequences. Ideally, good matching heuristics can assign or match a query against the correct reference with high precision while also retaining high recall (i.e., being sensitive to sequencing error or small divergence between the query and the reference). One particular type of alignment-free method for assigning reads to compatible references that has recently gained substantial traction is *pseudoalignment* [9–12]. While tremendous progress has been made in supporting alignment-free methods for metagenomic analyses, continued development of ever more efficient indexing methods is required for such analyses to scale to tens, even hundreds, of thousands of bacterial reference genomes.

A practical data structure that is suitable for alignment-free matching methods is the *colored de Bruijn graph*, a graph where each node corresponds to a $k$-mer in a reference collection and is annotated with a *color*—the set of references in which it occurs. Representing this data structure in small space while supporting efficient retrieval of the color of any $k$-mer is the goal of this work. An overview of our contributions is given below.

### Contributions

- Conceptually, our data structure arises as the composition of a $k$-mer dictionary with a compressed inverted index: the dictionary represents all $k$-mers in the indexed collection, whereas the inverted index explicitly stores all distinct colors (sorted integer sequences). We show how this two-level layout can be implemented in very compact space while granting efficient random and streaming queries, by leveraging recent advancements in order-preserving $k$-mer dictionaries [13, 14]. Specifically, we exploit the order-preserving property of the $k$-mer dictionary SSHash [13, 14] to store the $k$-mers in color order, so that consecutive $k$-mers have the same color. This allows the construction of a map from $k$-mers to colors in just $1+o(1)$ bits per unitig (i.e., unary paths) of the underlying colored compacted de Bruijn graph. To further reduce space, our index makes use of a simple but effective hybrid compression scheme for the colors.
  An important consequence of using SSHash as $k$-mer dictionary is that our index also supports very fast streaming queries for consecutive $k$-mers in a read, and additionally allows efficient implementation of skipping heuristics that have previously been suggested to speed up pseudoalignment [9].
- We implemented our index in a C++ tool called Fulgor, which is available at https://github.com/jermp/fulgor.
- We extensively compare Fulgor against the state of the art. Compared to Themisto  [15] built with default parameters Fulgor indexes a collection of 150,000 *Salmonella Enterica* genomes in $43\%$ less space, is at least twice as fast at query time, and even twice as fast to construct. For a subset of 100,000 *Salmonella Enterica* genomes, the largest collection that we could index with MetaGraph’s most space efficient variant (*row-diff* “relaxed” BRWT), MetaGraph requires $10\times$ less disk space but is also $20\times$ slower to query, much slower to construct than Fulgor, and requires significantly more memory to query (a more thorough discussion of MetaGraph’s additional optimization that trades-off increased memory usage for improved query speed can be found in "Query speed" section).
- Perhaps unsurprisingly, the rapid development of novel indexing data structures has been accompanied by novel and custom strategies for matching and assigning reads to colors (i.e., reference sets) and algorithms that each make different design choices and trade-offs. Many of these strategies can be considered as a form of pseudoalignment. Having been iterated on since its introduction [9], the term “pseudoalignment” has come to describe a family of efficient heuristics for read-to-color assignment, rather than a single concept or algorithm. Prior methods have taken either *exhaustive* approaches that queries every $k$-mer on a read (previously termed *exact* pseudoalignment  [12, 15]) or have implemented *skipping* based approaches that skip the query of “redundant” consecutive $k$-mers that likely map to the same set of reference genomes [9, 16]. To our knowledge, the precise details of the types of skipping heuristics used in the latter methods—including those adopted by the initial pseudoalignment method—have been discussed only in passing. Complete details, instead exist only in the source code of the corresponding tools. To shed light on these algorithms, we provide a more structured discussion of how these algorithms are designed. Using Fulgor, we implement two previously proposed variants and benchmark them.

## Preliminaries

In this section, we first formalize the problem under study here. We then describe a modular indexing layout that solves the problem using the interplay between two well-defined data structures. Lastly we describe the properties induced by the problem and how these are elegantly captured by the notion of *colored compacted de Bruijn graph*.

### Problem definition

#### Problem 1

(Colored *k*-mer indexing problem) Let $\mathcal {R}=\{R_1,\ldots ,R_N\}$ be a collection of references. Each reference $R_i$ is a string over the DNA alphabet $\Sigma =\{A,C,G,T\}$. We want to build a data structure (referred to as the *index*) that allows us to retrieve the set $\textsc {Color}{(x)} =\{i | x \in R_i\}$ as efficiently as possible for any $k$-mer $x \in \Sigma ^k$. Note that $\textsc {Color}{(x)} =\varnothing$ if *x* does not occur in any reference.

Hence, we call the set $\textsc {Color}{(x)}$ the *color* of the $k$-mer *x*.

### Modular indexing layout

In principle, Problem 1 could be solved using an old but elegant data structure: the *inverted index* [17, 18]. The inverted index, say $\mathcal {L}$, stores explicitly the ordered set $\textsc {Color}{(x)}$ for each $k$-mer $x \in \mathcal {R}$. What we want is to implement the map $x \rightarrow \textsc {Color}{(x)}$ as efficiently as possible in terms of both memory usage and query time. To this end, all the distinct $k$-mers of $\mathcal {R}$ are stored in a dictionary data structure, $\mathcal {D}$. Suppose the dictionary $\mathcal {D}$ stores *n* $k$-mers. To implement the map $x \rightarrow \textsc {Color}{(x)}$, the operation that $\mathcal {D}$ is required to support is $\textsc {Lookup}{(x)}$ which returns $\bot$ if $k$-mer *x* is not found in the dictionary or a unique integer identifier in $[n]=\{1,\ldots ,n\}$ if *x* is found. Problem 1 can then be solved using these two data structures—$\mathcal {D}$ and $\mathcal {L}$—thanks to the interplay between $\textsc {Lookup}{(x)}$ and $\textsc {Color}{(x)}$: logically, the index stores the sets $\{\textsc {Color}{(x)} \}_{x\in \mathcal {R}}$ in compressed format in the order given by $\textsc {Lookup}{(x)}$.

To our knowledge, all prior solutions proposed in the literature that fall under the “color-aggregative” classification [19], are incarnations of this *modular indexing framework* and, as such, require an efficient $k$-mer dictionary joint with a compressed inverted index. For example, Themisto  [15] makes use of the *spectral* BWT (or SBWT) data structure [20] for its $k$-mer dictionary, whereas MetaGraph [21] implements a general scheme to compress metadata associated to $k$-mers which is, in essence, an inverted index.

### The colored compacted de Bruijn graph and its properties

Problem 1 has some specific properties that one would like to exploit to implement as efficiently as possible the modular indexing framework described in "The colored compacted de Bruijn graph and its properties" section. First, consecutive $k$-mers share $(k-1)$-length overlaps; second, co-occurring $k$-mers have the same color. A useful, standard, formalism that describes these properties is the *colored compacted de Bruijn graph* (abbreviated “ccdBG”).

Given the collection of references $\mathcal {R}$, the (node-centric) de Bruijn graph (dBG) of $\mathcal {R}$ is a directed graph whose nodes are all the distinct $k$-mers of $\mathcal {R}$ and there is an edge connecting node *u* to node *v* if the $(k-1)$-length suffix of *u* is equal to the $(k-1)$-length prefix of *v*. We refer to $k$-mers and nodes in a (node-centric) dBG interchangeably; likewise, a path in a dBG spells the string obtained by “glueing” together all the $k$-mers along the path. Thus, unary (i.e., non-branching) paths in the graph can be collapsed into single nodes spelling strings that are referred to as *unitigs*. The dBG arising from this compaction step is called the compacted dBG (cdBG). Lastly, the *colored* compacted dBG is obtained by logically annotating each $k$-mer *x* with its color, $\textsc {Color}{(x)}$, and only collapsing non-branching paths with nodes having the same color.

Below, we notate *n* to be the number of distinct $k$-mers of $\mathcal {R}$ and *m* to be the number of unitigs $\{u_1,\ldots ,u_m\}$ of the ccdBG induced by the $k$-mers of $\mathcal {R}$. The unitigs of the ccdBG that we consider have the following key properties.

1. *Unitigs are contiguous subsequences that spell references in* $\mathcal {R}$. Each distinct $k$-mer of $\mathcal {R}$ appears once, as sub-string of some unitig of the cdBG. By construction, each reference $R_i \in \mathcal {R}$ can be a *tiling* of the unitigs—a sequence of unitig occurrences that spell out $R_i$ [22]. Joining together $k$-mers into unitigs reduces their storage requirements. In "The *k*-mer dictionary: mapping *k*-mers to unitigs with SSHash" and "Mapping unitigs to colors" sections, we show how this property can be exploited to make indexes compact. In "Pseudoalignment algorithms" section, we show how this property can be exploited to make queries fast.
2. *Unitigs are monochromatic*. The $k$-mers belonging to the same unitig $u_i$ all have the same color. Thus, we shall use ${\textsc {Color}{(u_i)}}$ to denote the color of each $k$-mer $x \in u_i$. We note that this property holds only if one considers $k$-mers appearing at the start or end of reference sequences to be *sentinel* $k$-mers that must terminate their containing unitig [23–25], and that such conventions are not always adopted [26, 27].
3. *Unitigs co-occur and share colors.* Unitigs often have the same color (i.e., occur in the same set of references) because they derive from conserved sequences in indexed references that are longer than the unitigs themselves. We indicate with *M* the number of distinct color sets $\mathcal {C}=\{C_1, \dots , C_M\}$. Note that $M \le m$ and that in practice there are dramatically more unitigs than there are distinct colors. We use ${\textsc {Color-ID}{(u_i)}} = j$ to indicate that unitig $u_i$ has color $C_j$. As a consequence, each $k$-mer $x \in u_i$ has color $C_j$.

In this work our goal is to design an index that takes full advantage of these key properties.

## Index description

In this section we describe a modular index that implements a colored compacted de Bruijn graph (ccdBG) and fully exploits its properties described in "The colored compacted de Bruijn graph and its properties" section. We adopt the modular indexing framework from "Modular indexing layout" section—comprising a $k$-mer dictionary $\mathcal {D}$ and an inverted index $\mathcal {L}$—to work seamlessly over the *unitigs* of the ccdBG. We extend the ideas from Fan et al. [22] for the modular indexing of $k$-mer positions to $k$-mer colors.

**Fig. 1:**
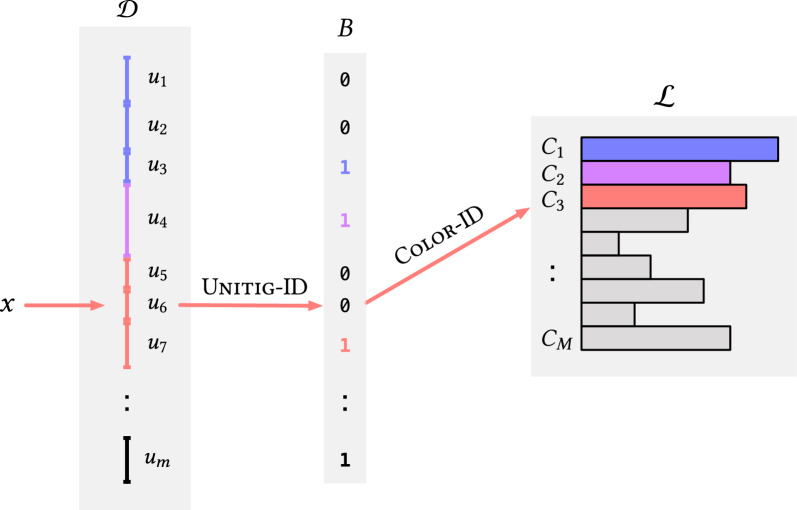
A schematic picture of the index described in "Index description" section, highlighting the interplay between the $k$-mer dictionary $\mathcal {D}$, the bit-vector *B*, and the inverted index $\mathcal {L}$. The red arrows show how the index is queried for a $k$-mer *x*, assuming that *x* occurs in unitig $u_6$ and has color $C_3$. The $k$-mer *x* is first mapped by $\mathcal {D}$ to its unitig $u_6$ via the query $\textsc {Unitig-ID}{(x)} =6$. Then we compute ${\textsc {Color-ID}{(u_6)}}=\textsc {Rank}_1(6,B)+1=2+1=3$ and lastly retrieve $C_3$ from $\mathcal {L}$

Our strategy is to first map $k$-mers to unitigs using a dictionary $\mathcal {D}$, and then map unitigs to their colors $\mathcal {C} =\{C_1,\ldots ,C_M\}$. By *composing* these mappings, we obtain an efficient map directly from $k$-mers to their associated colors. The colors themselves in $\mathcal {C}$ are stored in compressed form in a inverted index $\mathcal {L}$. Figure 1 offers a pictorial overview of how we orchestrate these different components in the index. The goal of this section is to describe how these mapping steps can be performed efficiently and in small space.

### The $k$-mer dictionary: mapping $k$-mers to unitigs with SSHash

For a $k$-mer dictionary, we use the SSHash data structure [13, 14], which fulfills the requirement described in "Modular indexing layout" section, in that it implements the query $\textsc {Lookup}{(x)}$ for any $k$-mer *x* efficiently and in compact space. This is achieved by storing the unitigs explicitly (i.e., as contiguous, 2-bit encoded strings) in some prescribed order so that a $k$-mer *x* occurring in some unitig $u_i$ can be quickly located using a minimal perfect hash function [28] built for the set of the *minimizers* [29] of the $k$-mers. Laying out unitigs in this principled manner also enables very efficient streaming query. That is, when querying consecutive $k$-mers from input reads, the query for a given $k$-mer can often be answered very efficiently given the query result from its predecessor, since it often shares the same minimizer and frequently even occupies the very next position on the same unitig as its predecessor. We refer the interested reader to [13, 14] for a complete overview of SSHash.

Even more importantly for our purposes, a query into the SSHash dictionary returns, among other quantities, $\textsc {Unitig-ID}{(x)} =i$, the ID of the unitig containing the $k$-mer *x*, as a byproduct of $\textsc {Lookup}{(x)}$. For any $k$-mer occurring in $\mathcal {R}$, $\textsc {Unitig-ID}{(x)} =i$ is an integer in [1..*m*]. This map from $k$-mers to unitigs will be exploited in the subsequent sections.

### Mapping unitigs to colors

Now that we have an efficient map from $k$-mers to unitigs, i.e., the operation $\textsc {Unitig-ID}{(x)}$, we must subsequently map unitigs to distinct colors. That is, we have to describe how to implement the operation ${\textsc {Color-ID}{(u_i)}}$ for each unitig $u_i$. Since each ${\textsc {Color-ID}{(u_i)}}$ is an integer in [1..*M*], we could implement ${\textsc {Color-ID}{(u_i)}}$ just by storing ${\textsc {Color-ID}{(u_1)}},\ldots ,{\textsc {Color-ID}{(u_m)}}$ explicitly in an array of $\lceil \log _2(M)\rceil$-bit integers. We show how to do this in just $1+o(1)$ bits per unitig rather than $\lceil \log _2(M)\rceil$ bits per unitig.

We do so by exploiting another key property of SSHash: the unitigs it stores internally can be permuted in any desired order without impacting the correctness or efficiency of the dictionary. This was already noted and exploited in [14] to compress $k$-mer abundances. Similarly, here we sort the unitigs by ${\textsc {Color-ID}{(u_i)}}$, so that all the unitigs having the same color are stored consecutively in SSHash. To compute ${\textsc {Color-ID}{(u_i)}}$, all that is now required is a $\textsc {Rank}_1$ query over a bit-vector *B*[1..*m*] where:

- $B[i]={\texttt {1}}$ if ${\textsc {Color-ID}{(u_i)}} \ne {\textsc {Color-ID}{(u_{i+1})}}$ and $B[i]={\texttt {0}}$ otherwise, for $1 \le i < m$;
- $$B[m]={\texttt {1}}$$.

It follows that *B* has exactly *M* bits set. The operation $\textsc {Rank}_1(i,B)$ returns the number of ones in *B*[1, *i*) and can be implemented in *O*(1) time, requiring only *o*(*m*) additional bits as overhead on top of the bit-vector [30, 31]. This means that ${\textsc {Color-ID}{(u_i)}}$ can be computed in *O*(1) as $\textsc {Rank}_1(i,B)+1$.

We illustrate this unitig to color ID mapping in Fig. 1. In this toy example, ${\textsc {Color-ID}{(u_6)}} = 3$ can be computed with $\textsc {Rank}_1(6,B) + 1 = 2 + 1$ because there are two bits set in *B*[1, 6)—each marking where previous groups of unitigs with the same color end. Therefore, according to *B*, unitigs $\{u_1,u_2,u_3\}$ all have the same color as also $\{u_5,u_6,u_7\}$; $u_4$’s color is not shared by any other unitig instead.

### Compressing the colors

The inverted index $\mathcal {L}$ is a collection of sorted integer sequences $\{C_1,\ldots ,C_M\}$, whose integers are drawn from a universe of size *N* (the total number of references in the collection $\mathcal {R}$). There is a plethora of different methods that may be used to compress integer sequences (see, e.g., the survey [18]). Testing the many different techniques available on genomic data is surely an interesting benchmark study to carry out. Here, however, we choose to adopt a simple strategy based on the widespread observation that effective compression appears to require using different strategies based on the density of the sequence $C_i$ to be compressed (ratio between $|C_i|$ and *N*) [18]. For example, for the colored $k$-mer indexing problem, Alanko et al. also observe and report highly skewed distributions of color densities [15].

We therefore implement the following *hybrid* compression scheme:

1. For a sparse color set $C_i$ where $|C_i|/N < 1/4$, we adopt a delta-gap encoding: the differences between consecutive integers are computed and represented via the universal Elias’ $\delta$ code [32].
2. For a dense color set $C_i$ where $|C_i|/N > 3/4$, we first take the complementary set of $C_i$, that is, the set $\overline{C_i}=\{j \in [1..N]| j \notin C_i\}$, and then compress $\overline{C_i}$ as explained in 1. above.
3. Finally, for a color set $C_i$, that does not fall into either above density categories, we store a characteristic bit-vector encoding of $C_i$—a bit-vector *b*[1..*N*] such that $b[j] = {\texttt {1}}$ if $j \in C_i$ and $b[j]={\texttt {0}}$ otherwise.

The compressed representations of all sequences are then concatenated into a single bit-vector, say *sequences*. An additional sorted sequence, $offsets [1..M]$, is used to record where each sequence begins in the bit-vector *sequences*, so that the compressed representation of the *i*-th sequence begins at the bit-position $offsets [i]$ in $sequences$, $1 \le i \le M$. The *offsets* sequence is compressed using the Elias-Fano encoding [33, 34] and takes only a (very) small part of the whole space of $\mathcal {L}$ unless the sequences are very short.

This hybrid encoding scheme is similar in spirit to the one also used in Themisto which, in turn, draws inspiration from Roaring bitmaps [35]. However, our choice of switching to the complementary set when $|C_i|$ approaches *N* turns out to be a very effective strategy, especially for pan-genome data, where a striking fraction of integers in $\mathcal {L}$ are indeed covered by these extremely dense sets (see also Table 4 from "Results" section).

### Construction

Fulgor is constructed by directly processing the output of GGCAT [27], an efficient algorithm to build ccdBGs using external memory and multiple threads. Importantly, GGCAT provides the ability to iterate over unitigs grouped by color. Therefore, Fulgor construction just requires a single scan of the unitigs in the order given by GGCAT. SSHash is built on the set of unitigs, each distinct color is compressed as described in "Compressing the colors" section, and the bit-vector *B* is also built during the scan.

**Algorithm 1:**
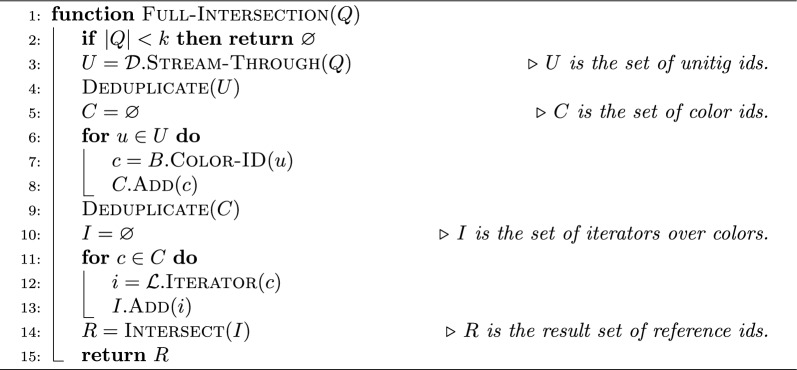
The $\textsc {Full-Intersection}$ algorithm for a query sequence *Q*. The algorithm uses the three index components: $\mathcal {D}$ (the dictionary, mapping $k$-mers to unitigs), *B* (the bit-vector mapping from unitigs to colors), and $\mathcal {L}$ (the inverted index storing the compressed colors). As discussed in Section , the dictionary $\mathcal {D}$ can stream through the query sequence *Q* and collect unitig ids. The inverted index $\mathcal {L}$, instead, returns an iterator over a color set given the color id *c* as $\textsc {Iterator}{(c)}$

**Algorithm 2:**
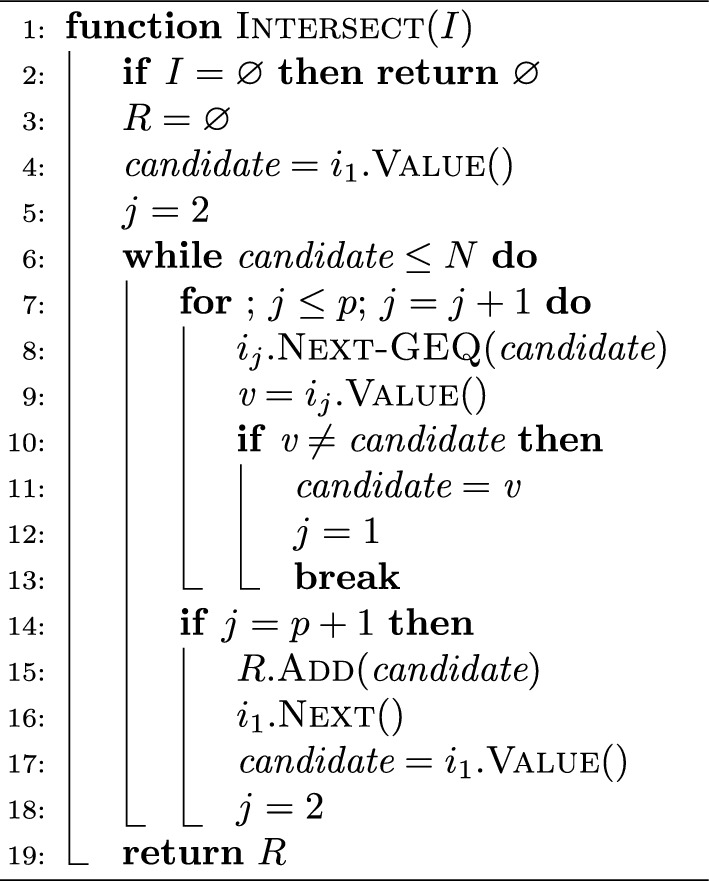
The $\textsc {Intersect}$ algorithm for a set of iterators $I=\{i_1,\ldots ,i_p\}$. An iterator object supports three primitive operations: $\textsc {Value}{()}$, returning the value currently pointed to by the iterator; $\textsc {Next}{()}$, returning the value immediately after the one currently pointed to by the iterator; $\textsc {Next-GEQ}{(x)}$, returning the smallest value that is larger-than or equal-to *x*. We assume that if *i* is an iterator over color $C_j$ then calling $i.\textsc {Next}{()}$ for more than $|C_j|$ times will return the (invalid) reference id $N+1$

## Pseudoalignment algorithms

The term *pseudoalignment*, originally coined by Bray et al. [9] and developed in the context of RNA-seq quantification, has been used to describe many different algorithms and approaches, several of which do not actually comport with the original definition. Specifically, Bray et al. [9] define a “pseudoalignment of a read to a set of transcripts, *T*” as “a subset, $S \subseteq T$, without specific coordinates mapping each base in the read to specific positions in each of the transcripts in *S*”. The goal of such an approach then becomes to determine, for a given read, the *set* of indexed reference sequences with which the read is *compatible*. In the most basic scenario, the compatibility relation can be determined entirely by the presence/absence of $k$-mers in the read in specific references.

Given any index of $k$-mer colors, a variety of different pseudoalignment algorithms can be implemented that rapidly map reads to compatible reference sequences according to a set of heuristics. Below, we review four pseudoalignment algorithms and describe their properties. Various existing tools implement a subset of these pseudoalignment strategies; we describe how Fulgor implements *all four* and give the corresponding pseudocodes.

The pseudoalignment algorithms we describe in this section fall into two categories:

1. *exhaustive* methods that retrieves the color of every $k$-mer on a given read (as described in [15]), and
2. *skipping* heuristics that skip or jump over $k$-mers during pseudoalignment that are likely to be *uninformative* (i.e., to have the same color as the $k$-mer that was just queried).

### Exhaustive methods

For a given query sequence *Q*, exhaustive approaches return colors with respect to a set of $k$-mers of *Q*, *K*(*Q*), that map to a non-empty color (i.e., each $k$-mer $x \in K(Q)$ if found in the dictionary $\mathcal {D}$).

**full-intersection** The first of the two exhaustive approaches, the *full-intersection* method, simply returns the intersection between all the colors of the $k$-mers in *K*(*Q*). Algorithm 1 shows how this query mode is implemented in Fulgor. In the current implementation, Fulgor has a generic intersection algorithm that can work over *any* compressed color sets, provided that an iterator over each color supports two primitives—next and $\textsc {Next-GEQ}{(x)}$, respectively returning the integer immediately after the one currently pointed to by the iterator and the smallest integer which larger-than or equal-to *x*. (We point the reader to [36] and [18] for details.)

**threshold-union** The second algorithm, which we term the *threshold-union* approach, relaxes the full-intersection method to trade off precision for increased recall. Instead of requiring a reference to be compatible with *all* mapped $k$-mers, the threshold-union method requires a reference to be compatible with a user defined proportion of $k$-mers. Given a parameter $\tau \in (0,1]$, this method returns the set of references that occur in *at least* $s \cdot \tau$ returned (i.e., non-empty) $k$-mer colors, where *s* can be either chosen to be $s=|K(Q)|$ (the number of positive $k$-mers only) or $s=|Q|-k+1$ (the total number of $k$-mers in *Q*). Themisto [15] implements the variant with $s=|K(Q)|$ (called the “hybrid” method), whereas both Bifrost [26] and MetaGraph  [21] use $s=|Q|-k+1$. In fact, the latter approach of simply looking up all of the $k$-mers in a query, and requiring a specified fraction of them to match, is a long-standing strategy that predates the notion of pseudoalignment [3, 37]. In the following, we assume $s=|K(Q)|$ is used by the threshold-union algorithm, unless otherwise specified. The pseudocode for this query mode is given in Algorithm 3.

In practice, both the aforementioned exhaustive methods are efficient to compute for two reasons. First, intersections, thresholding, and unions are easy to compute because colors are encoded as monotonically increasing lists of reference IDs. Second, for Fulgor in particular, querying *every* $k$-mer for its color can be performed in a highly-optimized way via *streaming* queries to SSHash. In the streaming setting, SSHash may skip comparatively slow hashing and minimizer lookup operations because it stores *unitig* sequences contiguously in memory. When sequentially querying adjacent $k$-mers on a read that are also likely adjacent on indexed unitigs, it can rapidly lookup and check $k$-mers that are cached and adjacent in memory (we refer the reader to [13] for more details).

### *Skipping* heuristics

**Fig. 2:**
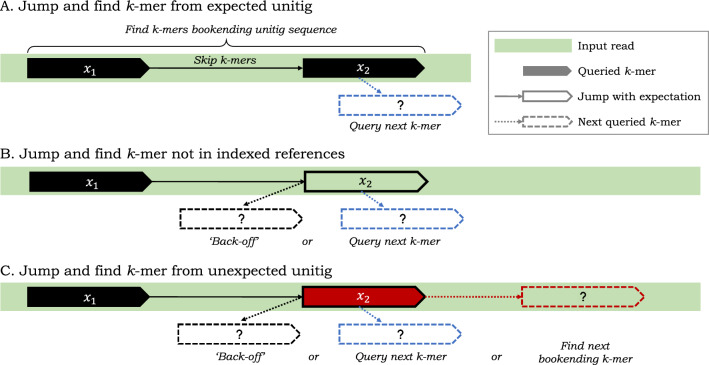
Some relevant design choices for pseudoalignment with skipping heuristics that *jump* and skip $k$-mers on a given read. After $k$-mer $x_1$ is queried and found to map to a “black” unitig, an algorithm can jump to query the $k$-mer $x_2$ on input read, where the number of $k$-mers skipped is given by the length of the black unitig. **A** In the ideal scenario, $x_2$ maps to the black unitig sequence and $k$-mers $x_1$ and $x_2$ are found to bookend this unitig sequence as it appears on the read. **B** If $x_2$ misses the index, an algorithm can *back-off* to an earlier $k$-mer on the read to find a $k$-mer bookending a shorter subsequence of the black unitig; or it may just query the next $k$-mer. **C** If $x_2$ maps to a different “red” unitig, an algorithm has an alternative, aggressive, heuristic option to jump and find the next $k$-mer bookending the red unitig sequence

For even faster read mapping, pseudoalignment algorithms can implement heuristic *skipping* approaches that avoid exhaustively querying all $k$-mers on a given read. These skipping heuristics make the assumption that whenever a $k$-mer on a read is found to belong to a unitig, subsequent $k$-mers will likely map to the same unitig and can therefore be skipped, since they will be uninformative with respect to the final color assigned to the query (i.e., the intersection of the colors of the mapped $k$-mers).

Bray et al. [9] first described such an approach, where a successful search that returns a unitig *u* triggers a skip that moves the search position forward to either the end of the query or the implied distance to the end of *u* (whichever is less). Subsequent searches follow the same approach as new unitigs are discovered and traversed in the query. Later, other tools extended or modified the proposed skipping heuristics, and introduced “structural constraints”, which take into account the co-linearity and spacing between matched seeds on the query and on the references to which they map [16]. In contrast to Themisto, Fulgor has rapid access to the topology of the ccdBG because its $k$-mer dictionary, SSHash, explicitly maps $k$-mers to unitig sequences that are stored contiguously in memory. Fulgor thus permits efficient implementation of pseudoalignment algorithms with skipping heuristics since, due to the underlying capabilities provided by SSHash, it can rapidly find $k$-mers bookending unitig substrings because SSHash can explicitly map $k$-mers to their offsets (positions) in indexed unitig sequences.

**Algorithm 3:**
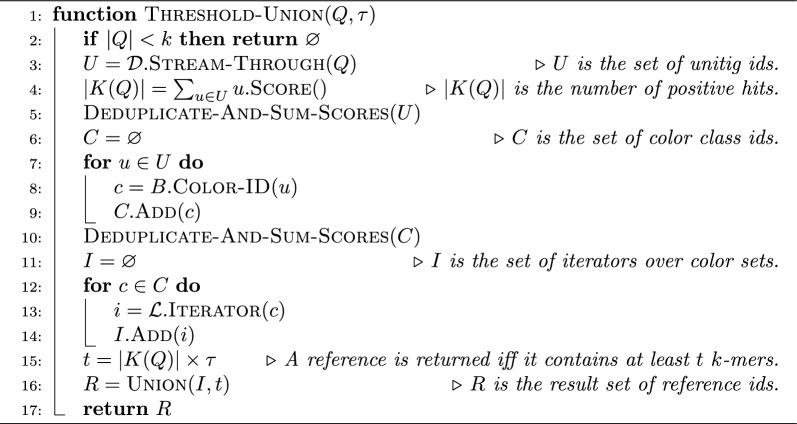
The $\textsc {Threshold-Union}$ algorithm for a query sequence *Q*. Differently from the full-intersection method (Algorithm 1), here *U*, *C*, and *I*, are sets of pairs. The first component of a pair is a unitig id, a color id, or an iterator, respectively if the pair is in *U*, *C*, or *U*. The second component, read by calling the method $\textsc {Score}{()}$ in the pseudocode, is the number of positive $k$-mers that have a given unitig id or have a given color. The score of iterator *i* is the score of the color id *c* if $i=\mathcal {L}.\textsc {Iterator}{(c)}$. Clearly, when deduplicating the sets *U* and *C*, the scores of equal unitig or color ids must be summed

**Algorithm 4:**
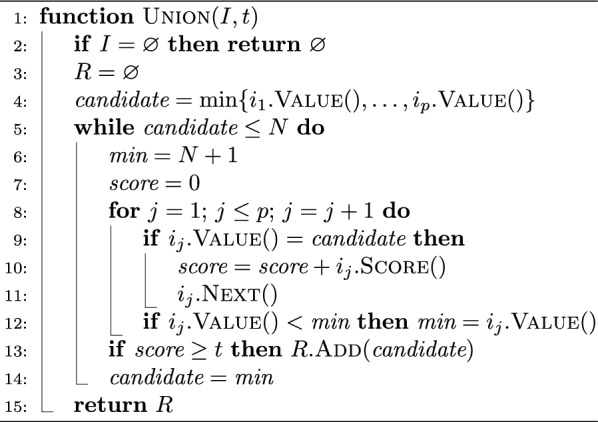
The $\textsc {Union}$ algorithm for a set of iterators $I=\{i_1,\ldots ,i_p\}$ and minimum score *t*.

In general, pseudoalignment methods that implement skipping heuristics must specify what steps the algorithm will take in *all* scenarios, not just what should happen when search proceeds as expected. In practice, implementations for resolution strategies are complicated and difficult to describe succinctly in prose, and prior work has only discussed these important details in passing. Here, using the depicted scenarios in Fig. 2, we provide a more structured (though certainly not exhaustive) discussion of possible design choices that can be made. These design choices impact the performance of the pseudoalignment algorithm, both in terms of how many $k$-mers it queries (and, hence, its speed), and in how many distinct color sets it collects (and, hence, the actual compatibility assignment it makes).

**Jump and find** *k*-**mer in expected unitig** Before the first matching $k$-mer of a read is found, there is relatively little difference between exhaustive and heuristic pseudoalignment approaches; subsequent $k$-mers are queried until the read is exhausted or some $k$-mer is found in the index. At this point, however, heuristic skipping methods diverge from the exhaustive approaches. At a high level, when a $k$-mer on a read is found to map to a unitig, skipping heuristics make an assumption that said unitig appears wholly on the read. A pseudoalignment algorithm then jumps, on the read, to what would be the last $k$-mer on the unitig sequence occurring on the given read (i.e., a bookending $k$-mer). Scenario A in Fig. 2 depicts when this assumption is correctly made. Moving left-to-right on a given read, if a $k$-mer on the *left* is found to occur on the unitig depicted in black color in the figure (referred to as the “black” unitig henceforth), an algorithm can then skip a distance given by the length of the black unitig and jump to a $k$-mer to the right that also maps to the black unitig and bookends it. Doing so, an algorithm can assume that all $k$-mers bookended by these two queried $k$-mers map to the black unitig, avoid querying $k$-mers in-between, and instead continue to query the next $k$-mer on the read (indicated in dashed lines in blue).

**Jump and miss** *k*-**mer** In practice however, the implemented skipping heuristics are not so simple. This is because, when skipping $k$-mers according to unitig lengths, the resulting $k$-mer that an algorithm jumps to may not necessarily map to the unitig it *expects*. In scenario B, an algorithm jumps to a $k$-mer on a read, expecting it to map to a black unitig, but finds that it does not correspond to any indexed $k$-mer. Here, an algorithm can make several choices, and in fact, current skipping heuristics make two distinct choices in this scenario. It can ignore this missed $k$-mer and simply query the next $k$-mer after the position that was jumped to (in blue). Or, it can take a more conservative approach and implement a *back-off* scheme to look for another $k$-mer that maps to the black unitig. An algorithm can back-off and jump a lesser distance, and such a back-off approach can happen once or can be recursive or iterative until some termination condition is satisfied.

**Jump and find** *k*-**mer in un-expected unitig** In scenario C, an algorithm that jumps to a $k$-mer but finds that it maps to a *different* (red) unitig than expected. Here, we suggest three choices an algorithm can make. Like in scenario B, an algorithm can back-off to find another $k$-mer mapping to the black unitig or it can query the next $k$-mer after the jumped position. Alternatively, it can take a new more aggressive approach and jump to a $k$-mer on the read where it expects to find the end of an occurrence of the red unitig.

In this work, we have retrofitted the pseudoalignment with skipping algorithms from Kallisto [9]^1^ and Alevin-fry [16]^2^ to make use of Fulgor, rather than the distinct indexes atop which they were implemented in their original work. Using Fulgor, we compare their resulting pseudoalignments, along with those from the full-intersection and threshold-union approaches, in a simple simulated scenario in "Comparison of pseudoalignment algorithms on simulated data" section.

### Multi-query optimizations

In addition to the many ways in which the actual mapping or pseudoalignment can be performed, it is also possible to further optimize query throughput—potentially at the cost of latency—by taking advantage of similarity among the query sequences themselves. One example of such an optimization is the *batch mode* of MetaGraph [21], which considers “batches” of query sequences for which it builds a query graph to exploit shared $k$-mers among the queries and mitigate the cost of color lookup and decoding in the index. On the other hand, the color-id query and even decoding the color itself are very fast operations for Fulgor. However, regardless of what index is being used to retrieve the colors of specific $k$-mers, the process of intersecting “large” colors (i.e., colors containing many references) can be a bottleneck to query. This problem is particularly pronounced when there are many similar references, and individual queries may return many reference labels.

Here, we devise and implement a simple scheme to accelerate query throughput, and note in "Conclusions and future work" section.  some interesting directions in which these ideas may be extended. Specifically, we develop a two-pass pseudoalignment algorithm for the full-intersection variant of pseudoalignment (though the ideas are extendable to other variants as well). The algorithm is motivated by two particular observations. First and as already observed above, retrieving color ids via the the color-id query is *very* fast—faster than retrieving and decoding the colors themselves. Second, in a sufficiently large collection of queries, many queries will share identical lists of color ids. Of course, it follows that any queries that share the same set of color ids will result in the same pseudoalignment, as the result of intersecting the colors associated with these ids will be the same.

**Fig. 3:**
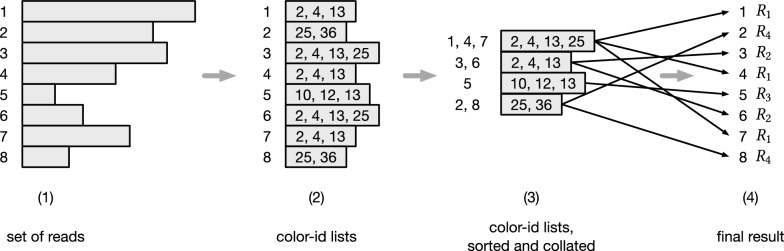
An example of the two-pass query optimization on a set of 8 query reads (1), that are assigned ids from 1 to 8. First, the color-id lists are generated from the input reads (2), then sorted and collated (3) so that the set of read ids having the same color-id list is retained for each distinct list. For example, the reads 1, 4, and 7 all have the same color-id list [2, 4, 13, 25]. Lastly, intersections are performed for each color-id list and each original read is annotated with its result (4). We have four distinct color-id lists in the example, hence four results $R_1$, $R_2$, $R_3$, and $R_4$ are computed. In the picture, the black thin arrows from (3) to (4) logically show how each original read is associated to its result $R_i$, $i=1,\ldots ,4$

Based on these observations, our two-pass algorithm proceeds as follows. First, we generate the list of distinct color ids for each query read. Let it be called the *color-id list* of the read. This step is very fast, usually taking only a few seconds for the high-hit workload datasets evaluated in this paper (see "Query speed" section). Each color-id list is also associated with the id of the read from which it was generated. Then, the color-id lists are sorted. The sort places consecutively all identical color-id lists, so that it is easy to retain the set of read ids associated with each *distinct* color-id list. Lastly, in a second pass, the distinct color-id lists are processed (i.e., their corresponding colors decoded and intersected) and the mapping results for each read group are recorded. Refer to Fig. 3 for an example.

This approach has at least two distinct benefits. First, all reads sharing duplicate color-id lists are handled together: rather than having to intersect the colors corresponding to the color-id list a number of times equal to the number of reads labeled with these ids, we perform the intersection only once—in other words, exactly duplicated list intersections are avoided. Second, by virtue sorting the color-id lists, we tend to observe distinct lists that share common prefixes of color ids nearby in the order, potentially improving the caching behavior of color lookup. While this approach directly exploits only exact duplicate color lists, and induces some extra work in terms of sorting and aggregating the complete set of color lists, we observe that it generally reduces the overall query time, and in our experiments led to query performance improvements of up to $40\%$ (see "Query speed" section).

We briefly describe in section  some possible extensions and generalizations of these ideas as interesting future work.

## Results

In this section, we report experimental results to assess Fulgor ’s construction time/space, index size, and query speed. All results are for $k=31$.

Experiments were run on a machine equipped with Intel Xeon Platinum 8276 L CPUs (clocked at 2.20GHz), 500 GB of RAM running Ubuntu 18.04.6 LTS (GNU/Linux 4.15.0). Fulgor is written in C++ and available at https://github.com/jermp/fulgor. For the experiments reported here we use v1.0.0 of the software, compiled with gcc 11.1.0.

**Datasets** We follow the experimental methodology of Alanko et al. [15] and build Fulgor over subsets of *S. Enterica* genomes (up to 150,000 genomes) from [38]to demonstrate Fulgor ’s effectiveness when indexing collections of similar reference sequences. We also consider a collection of 3,682 *E. Coli* genomes from NCBI [39] and a heterogeneous collection of 30,691 genomes of bacterial species representative of the human gut [40] (as also benchmarked in our previous work [22]). We report some summary statistics for the indexed ccdBGs in Table 1. Links to download the datasets are provided in the section of this article headed “Availability of data and materials”.

**Competitors** Throughout the section, we compare Fulgor to the following indexes. We use the C++ implementations from the respective authors. All software was compiled with gcc 11.1.0. A link to the respective libraries on GitHub can be found in the References.

- Themisto [15]. This is the most recent ccdBG index. In their evaluation, Alanko et al. show that Themisto embodies a better space/time trade-off compared to other methods that build similarly capable indexes (namely Bifrost [26] and MetaGraph [21]). Specifically, we build Themisto indexes using option -d1 which disables the sampling of $k$-mer colors in the SBWT for better query efficiency, and with option -d20 for better space effectiveness (this option is now the recommended choice). We use Themisto’s default color set representation (i.e., without Roaring bitmaps). We use the shipped compiled binaries (v3.1.1).
- MetaGraph [21, 41]. This is a flexible and highly configurable framework for indexing of reference sequences and metadata associated to $k$-mers in a ccdBG. In this study, we benchmark against MetaGraph ’s most space-efficient variant—the *row-diff* “relaxed” BRWT [21]. In brief, MetaGraph achieves a highly compressed *on-disk* representation by encoding differences in metadata of adjacent nodes in the ccdBG. Since Alanko et al. [15] previously showed that Themisto is comparable in speed but is significantly more space efficient than MetaGraph ’s uncompressed “plain” variant, we choose not to benchmark against it. We built the indexes using a workflow that we wrote with the input from the MetaGraph authors, available at https://github.com/theJasonFan/metagraph-workflows.
- COBS [42]. This is an approximate ccdBG index, in the sense that the pseudoalignment results may contain some false positives, i.e., identifiers of references that are falsely reported as containing the query $k$-mers. COBS represents each reference with a Bloom filter, which is filled with all the $k$-mers in the reference. The Bloom filter matrix is logically inverted, hence obtaining an approximate color matrix. Being approximate, the method completely avoids the space consumption of an exact $k$-mer dictionary and the space is all spent by the approximate color matrix. Very importantly, COBS partitions the input collection into shards of references of roughly the same size prior to indexing. This permits to build Bloom filters of different sizes: filters belonging to different shards have a different number of bits allocated, hence saving space compared to the case where all references are represented with filters of the same size. At query time, however, a $k$-mer lookup has to be resolved by every shard and individual results combined. We build COBS indexes with default parameters, as recommended by the authors: each filter has a false positive rate of 0.3 and one hash function; each shard contains at most 1024 references.
  It is interesting to inspect the performance of an approximate method such as COBS in comparison to exact methods to see if and how approximation brings some performance advantages.

**Table 1:** Summary statistics for the tested collections. The row “Integers in colors” reports the total number of reference IDs that are required to encode all colors—i.e., the sum set sizes for all colors, $\sum _{i} |C_i|$

	*E. coli* (EC)	*S. Enterica* (SE)					Gut Bacteria (GB)
Genomes	3682	5000	10,000	50,000	100,000	150,000	30,691
Distinct colors ($\times 10^6$)	5.59	2.69	4.24	13.92	19.36	23.61	227.80
Integers in colors ($\times 10^9$)	5.74	5.77	15.68	133.49	303.53	490.04	10.04
$k$-mers in dBG ($\times 10^6$)	170.65	104.69	239.88	806.23	1,018.69	1,194.44	13,936.86
Unitigs in dBG ($\times 10^6$)	9.31	4.95	8.24	30.64	41.16	49.60	566.39

**Table 2:** Total index construction time (elapsed time) and GB of memory (max. RSS), as reported by /usr/bin/time with option -v, using 48 processing threads

	Fulgor		Themisto		MetaGraph		COBS	
	hh:mm	GB	hh:mm	GB	hh:mm	GB	hh:mm	GB
EC	00:06	16.89	00:19	17.18	00:46	149.38	00:03	6.39
SE-5K	00:04	12.91	00:11	12.97	00:47	190.99	00:09	8.13
SE-10K	00:09	23.60	00:25	23.58	01:50	218.76	00:17	16.15
SE-50K	01:13	43.76	02:32	96.00	14:16	^*^118.95	01:41	82.49
SE-10K	02:56	73.54	06:25	202.42	26:40	^*^103.99	02:37	83.79
SE-150K	04:36	136.94	10:00	323.10	—	—	04:54	159.31
GB	02:27	115.05	06:21	183.56	10:50	^*^99.54	00:22	17.08

The reported time includes the time to serialize the index on disk and, for Fulgor and Themisto, the time taken by GGCAT to build the ccdBG. We did not observe appreciable differences in space and memory usage when building indexes for Themisto with and without $k$-mer sampling, except on the Gut Bacteria collection where sampling is very beneficial. For this reason, we report its best time and memory usage, i.e., that for Themisto-d20. MetaGraph instances marked by ^*^ were capped to use 100 GB of memory because construction otherwise exceeds total available memory ($> 500$ GB) on our machine

**Fig. 4:**
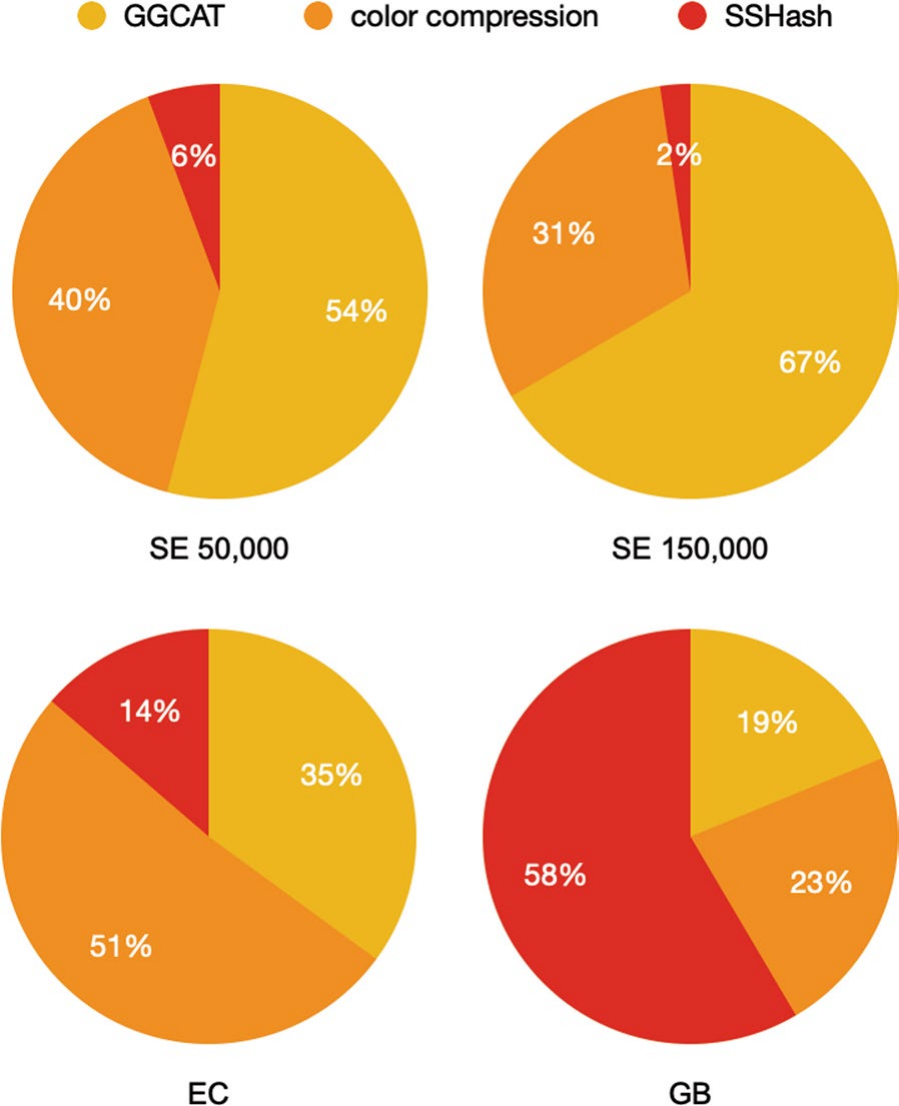
Construction time breakdown for Fulgor

### Construction time and space

Construction time and peak RAM usage is reported in Table 2 for the different datasets evaluated. Both Fulgor and Themisto use GGCAT to build the ccdBG. However, Fulgor is $2 - 6\times$ faster, and typically consumes much less memory during construction. This is because Themisto spends most of its time and memory building the color mapping. However, the analogous component of Fulgor is just a bit vector, demarcating groups of unitigs with the same color, that is built via a linear scan of the unitigs produced by GGCAT.

Figure 4 shows, instead, Fulgor’s construction time breakdown for some illustrative datasets. We distinguish between three phases in the construction: (1) running GGCAT, (2) compressing the colors and, (3) building SSHash. While GGCAT and color compression take most of the construction time on the Salmonella pangenomes, building SSHash is the most expensive step on the Gut Bacteria collection. This is consistent with the statistics reported in Table 1. Here, there are far more integers to compress in the Salmonella collections whereas the Gut Bacteria collection contains one order of magnitude more $k$-mers. This suggests that one could achieve even faster construction for Fulgor if the colors are compressed in parallel with the SSHash construction (currently, these two phases are sequential).

Compared to MetaGraph, Fulgor is faster to build across all benchmarked datasets. For example, on a collection of 10,000 Salmonella genomes, Fulgor is more than 12$\times$ faster to build. For datasets we were able to build MetaGraph with uncapped memory usage, Fulgor could be built with an order of magnitude less memory. It is important to note, however, that MetaGraph is likely doing more work than Fulgor in terms of compression as it achieves much smaller on-disk representations. Unfortunately, we were also unable to build MetaGraph instances on our largest datasets due to outsized memory and disk requirements for construction.

Compared to COBS, an *approximate* data-structure, Fulgor requires comparable time and memory to build (or even less memory on larger datasets) across all benchmarked datasets, except on GB. On the latter dataset, in fact, Fulgor spends 58% of its time in building SSHash (Fig. 4)—the exact $k$-mer dictionary component that COBS does not require entirely.

### Index size

The size of the indexes on disk is reported in Table 3. When indexing collections of Salmonella genomes, Fulgor is consistently $\approx 2\times$ smaller than Themisto-d1 and $\approx 1.8\times$ smaller than Themisto-d20. For example, on the largest collection comprising 150,000 genomes, Fulgor takes 70.66 GB whereas Themisto takes 133.63 GB and 126.74 GB (setting the sampling parameter -d to 1 and 20, respectively).

This remarkable space improvement is primarily due to the more effective color compression scheme adopted by Fulgor. This leads to, for example, 48% less space to encode colors for the 150,000 collection of Salmonella genomes. Looking at Table 4, we highlight that for all indexed Salmonella reference collections, approximately 50% of all encoded integers in the distinct colors belong to colors that are *at least* 90% dense. For such extremely dense colors, the complementary encoding strategy described in "Compressing the colors" section is very effective: only $\approx 0.2$ bits/int (bpi) are required to encode them in all benchmarked indexes. In fact, even for our largest collection of 150,000 Salmonella genomes, encoding *all* integers in *all* colors requires only 1.120 bpi.

**Table 3:** Index space in GB, broken down by space required for indexing the $k$-mers in a dBG (SSHash for Fulgor, SBWT for Themisto, and BOSS for MetaGraph); and data structures required to encode colors and map $k$-mers to colors

	Fulgor			Themisto-d1			Themisto-d20		MetaGraph			COBS
	dBG	Colors	Total	dBG	Colors	Total	Colors	Total	dBG	Colors	Total	Total
EC	0.29	1.36	1.65	0.22	2.75	2.97	1.85	2.08	0.10	0.23	0.33	7.53
SE-5K	0.16	0.59	0.75	0.14	1.82	1.96	1.29	1.43	0.07	0.19	0.26	9.11
SE-10K	0.35	1.66	2.01	0.32	4.78	5.09	3.50	3.81	0.13	0.38	0.51	18.68
SE-50K	1.26	17.03	18.30	1.07	36.89	37.96	32.42	33.48	0.36	1.95	2.31	88.61
SE-100K	1.72	40.70	42.44	1.35	81.82	83.17	75.94	77.28	0.45	3.50	3.95	173.58
SE-150K	2.03	68.60	70.66	1.58	132.05	133.63	125.16	126.74	—	—	—	265.49
GB	21.31	15.45	36.85	18.33	121.08	139.41	30.88	49.21	5.23	4.77	10.00	21.23

For COBS, we just report the total index size (that coincides with the colors’ space)

Unsurprisingly, Fulgor also uses less space than Themisto to support the $\textsc {Color-ID}$ operation. We recall from "Mapping unitigs to colors" section that Fulgor requires only $1+o(1)$ bits per unitig by design. This amounts to a negligible space usage compared to the overall index size. For example, while Themisto requires 7.26 GB to map $k$-mers to color IDs for 150,000 Salmonella genomes, our strategy just takes 7.75 MB.

When indexing a *heterogeneous* collection, e.g., the 30,691 bacterial genomes [40], with many more unique $k$-mers, the space advantage Fulgor has over Themisto is even more apparent. The overall size of Fulgor is $3.8\times$ smaller (36.77 GB versus 139.41 GB) than Themisto without sampling of $k$-mer colors (-d1). Here, Fulgor ’s near optimal approach of mapping *unitigs* to colors instead of $k$-mers to colors is dramatically more efficient, requiring only 88 MB compared to Themisto ’s 91GB. Themisto, by using the SBWT, organizes $k$-mers based on their *colexicographical* order and requires $\lceil \log _2(M)\rceil$ bits per sampled $k$-mer to record the color IDs. Here, the SBWT must record colors for each of the 13.9 billion distinct $k$-mers *and* their reverse complement. In contrast, Fulgor uses SSHash that maintains $k$-mers in unitig order and requires only $1 + o(1)$ bits per unitig to map *all* $k$-mers from the same unitig to a single color.

However, Themisto can improve its space usage by sampling $k$-mers. Note that Themisto built with -d20 is only 49.21 GB in size (compared to 139.41 GB without sampling). Fulgor is still smaller than Themisto-d20 by a large margin ($\approx 1.8\times$ on average). Especially on the Gut Bacteria collection, Themisto ’s sampling overcomes the inherent inefficiency of having to map each *individual* $k$-mer to a color

In contrast to Themisto, Fulgor efficiently maps each *unitig* (usually containing many $k$-mers) to a color—even after sampling $k$-mer colors, Fulgor encodes colors in almost half the space (15.45 GB versus 30.88 GB, respectively).

MetaGraph is the smallest on-disk representation. On 100,000 Salmonella genomes, the largest collection of genomes that we were able to index with MetaGraph, it requires $10\times$ less space than Fulgor. On the heterogeneous collection of 30,691 gut bacteria genomes, MetaGraph requires $3.7\times$ less space than Fulgor. However, as we are going to discuss in "Query speed" section, this remarkable space effectiveness comes at the price of a severe query time slowdown. (We were unable to build MetaGraph on the collection of 150,000 Salmonella genomes because of MetaGraph ’s outsized memory and external disk space requirements.)

**Table 4:** Average bits/int (bpi) spent for representing colors whose density is $(i \times 10)\%$ of *N*, for $i=1,\ldots ,10$

Density (%)	EC			SE						GB		
	$N=3,682$			$N=50,000$			$N=150,000$			$N=30,691$		
	lists	ints	bpi	lists	ints	bpi	lists	ints	bpi	lists	ints	bpi
$0-10$	46.72	3.90	7.96	70.96	2.62	6.00	79.23	3.27	6.32	99.99	99.99	12.05
$10-20$	11.11	5.85	5.24	3.74	2.84	3.05	2.54	2.68	3.92	0.00	0.00	0.00
$20-30$	8.05	7.10	4.01	2.69	3.50	3.24	2.09	3.76	3.46	0.00	0.00	0.00
$30-40$	4.95	6.14	2.90	1.90	3.43	2.89	1.40	3.51	2.88	0.00	0.00	0.00
$40-50$	4.56	7.34	2.23	1.81	4.25	2.22	1.29	4.19	2.23	0.00	0.00	0.00
$50-60$	3.85	7.55	1.84	1.82	5.24	1.82	1.24	4.94	1.82	0.00	0.00	0.00
$60-70$	4.29	10.00	1.54	2.04	6.94	1.53	1.40	6.59	1.54	0.00	0.00	0.00
$70-80$	4.20	11.23	1.33	2.33	9.13	1.08	1.64	8.91	1.15	0.00	0.00	0.00
$80-90$	3.65	11.12	0.91	3.03	13.43	0.56	2.09	12.95	0.66	0.00	0.00	0.00
$90-100$	8.63	29.73	0.31	9.67	48.63	0.19	7.07	49.21	0.21	0.00	0.00	0.00
Total bpi	1.893			1.020			1.120			12.32		

The first two columns for each collection, “lists” and “ints”, report the percentage of lists (i.e., colors) and integers (stored reference identifiers) that belong to all colors within a given density. The last row, “Total bpi”, is comprehensive of the space spent for the integer lists themselves and the space spent for the offsets delimiting the lists’ boundaries

### Query speed

To compare query speed, we benchmark Fulgor and Themisto using both low- and high-hit rate read-sets, i.e., read-sets for which we have a low and high number of positive $k$-mers respectively. Precisely, we use the files containing the first read of the following paired-end libraries in FASTQ format: SRR19282007^3^ with $7.3 \times 10^6$ reads, SRR896663^4^ with $5.7 \times 10^6$ reads, SRR801268^5^ with $6.6 \times 10^6$ reads, and ERR321482^6^ with $6.8 \times 10^6$ reads. For COBS, we report query times with the entire index loaded into RAM (option –load-complete.)

In Table 5 we report the result of the comparison using the full-intersection method (Algorithm 1). We repeated the same experiment using the threshold-union method (Algorithm 3) with parameter $\tau =0.8$ as this is the preferred query mode in Themisto and MetaGraph. However, we did not observe any appreciable difference compared to the full-intersection method in terms of query speed.

From a high-level point of view, the trend is as follows: Fulgor is consistently the fastest index to query, closely followed by Themisto, whereas both MetaGraph and COBS are much slower. We discuss details in the following.

In a low-hit rate workload where a small proportion of reads map to the indexed references, Fulgor is much faster than all benchmarked indexes. In this scenario, we expect many queried $k$-mers to not occur in the indexed references. When $k$-mers are absent from the index, no color needs to be retrieved and only the $k$-mer dictionary is queried. Here, Fulgor is faster than Themisto because its reliance on the fast streaming query capabilities of SSHash. It is worth noting here that in any *streaming* setting where consecutive $k$-mers are queried, Fulgor can fully exploit the monochromatic property of unitigs in ways which Themisto cannot. Queries to SSHash have very good locality compared to the SBWT because adjacent $k$-mers in unitigs are stored contiguously in memory. Further, streaming queries to SSHash can be very efficiently cached and optimized. When looking up consecutive $k$-mers, SSHash can entirely avoid computing its minimal perfect hash and instead perform fast comparisons of $k$-mers stored in cached positions pointing to adjacent addresses in memory.

In a high-hit rate workload, Fulgor still outperforms all benchmarked indexes, but outperforms Themisto by a smaller margin since most of the time is now spent in performing the intersection between colors. It is interesting to note that all indexes can process the workloads significantly faster on the Gut Bacteria collection: this is a direct consequence of the fact that the lists being intersected are much shorter on average for the Gut Bacteria compared to the Salmonella collections. This is evident from Table 4: essentially all lists are just 10% dense, i.e., have length at most $\lceil 30,691/10 \rceil < 3,070$.

For high-hit rate workloads, we also apply and benchmark Fulgor ’s two-pass query optimization (described in "Multi-query optimizations" section) in which *distinct* color id lists are first collected and intersected afterwards. This scheme ensures that, for a collection of queried reads, the intersection between distinct color id list is only performed *once*. From Table 6, we see that for high-hit rate workloads where identical color id lists are intersected frequently, this two-pass scheme consistently reduces total query times for *homogeneous* genome collections, while trading off additional memory usage. The fastest speedup we observe is from querying the Fulgor index of 50,000 Salmonella genomes where the two-pass scheme achieves a 40.7% speedup while using only 14% percent more memory. Unsurprisingly, the two-pass scheme is only effective when indexes contain many *similar* genomes. This is evident, for instance, on the heterogeneous collection of 30,691 gut bacteria genomes where the two-pass scheme does not significantly speed up queries albeit using 21.5% extra memory. In this case, deduplicating color id lists is not effective because most of them are already distinct.

Our results show interesting trade-offs and design choices in static vs. dynamic usage of memory at query time. Although small on-disk—in contrast to other methods where query-time memory usage closely matches on-disk size—MetaGraph requires more memory when retrieving metadata at query-time. For low-hit workloads, our results show that if minimizing space is the priority, MetaGraph, though slower than other methods, is the most memory frugal option. However, for high-hit workloads, MetaGraph in its fastest “batch” mode query, requires significantly more memory than the size of the index on-disk. For this workload and an index of 10,000 Salmonella genomes, a MetaGraph index requires only 0.51 GB of disk space but uses 92.18 GB of memory to query (hence, $180\times$ more memory). We were unable benchmark MetaGraph ’s batch mode queries for 50,000 and 10,000 genome collections because these experiments require more than 500 GB in memory.

We also note that part of the slowdown seen for Themisto is due to the time spent in loading the index from disk to RAM. For collections of Salmonella genomes, index load times are at least twice as Fulgor’s because of its larger index size. In Table 7, we measure the average loading time of the indexes from disk to memory (we have omitted MetaGraph from these measurements since MetaGraph ’s memory usage is dynamic and is not accurately reflected by its on-disk size, as explained above). Here, we can see that both Themisto (-d1) and COBS impose a non-negligible time overhead due to loading from disk. This impacts negatively on low-hit workloads where a significant fraction of the measured “query” time is spent in actually loading the index. So, while the result in Table 5 is fair since it reports the total query time end-to-end, Table 7 suggests that some indexes are only beneficial for heavy workloads where loading from disk is a smaller fraction of the total time. This is especially evident when comparing benchmarks on the collection of Gut Bacteria genomes for Themisto with and without $k$-mer color sampling. Here, the theoretically slower parameterization of Themisto (with -d20) completes benchmarks *faster* than a Themisto index without sampling because, though slightly slower to query, the smaller index is much faster to load.

**Table 5:** Total query time (elapsed time) and memory used during query (max. RSS) as reported by /usr/bin/time -v, using 16 processing threads

	Rate	Fulgor		Themisto-d1		Themisto-d20		MetaG.-B		MetaG.-NB		COBS	
		m:ss	GB	m:ss	GB	m:ss	GB	mm:ss	GB	mm:ss	GB	mm:ss	GB
(a) low-hit													
EC	4.71	0:10	1.65	0:33	3.22	0:33	2.35	7:34	2.82	3:40	0.38	10:25	28.94
SE-5K	1.27	0:09	0.77	0:32	2.27	0:30	1.77	6:48	2.76	2:55	0.31	11:50	37.64
SE-10K	13.86	0:10	2.01	0:36	5.32	0:36	4.06	7:35	3.00	4:17	0.56	14:33	75.63
SE-50K	32.61	0:25	17.91	1:05	37.45	0:56	33.07	8:33	5.05	6:47	2.42	39:33	367.34
SE-100K	34.09	0:45	41.49	1:39	81.60	1:22	75.89	9:19	7.04	7:33	4.23	48:52	^*^521.58
SE-150K	34.01	1:06	69.05	5:02	130.94	2:05	124.19	—	—	—	—	37:40	^*^522.47
GB	11.90	0:57	36.02	2:58	136.47	1:42	48.37	11:03	12.24	11:55	9.89	30:01	192.70

	Rate	Fulgor		Themisto-d1		Themisto-d20		MetaG.-B		MetaG.-NB		COBS	
		mm:ss	GB	h:mm:ss	GB	h:mm:ss	GB	mm:ss	GB	h:mm:ss	GB	h:mm:ss	GB
(b) high-hit													
EC	99.10	02:10	1.68	0:03:40	3.32	0:03:40	2.46	22:00	30.44	1:05:41	0.40	0:45:11	34.93
SE-5K	89.53	01:16	0.82	0:03:50	2.34	0:03:50	1.82	14:14	36:54	0:20:32	0.33	0:38:34	41.93
SE-10K	89.76	02:26	2.11	0:07:35	5.40	0:07:35	4.16	28:15	92.18	0:43:40	0.61	1:01:14	84.20
SE-50K	91.31	19:15	18.53	0:41:25	37.52	0:42:02	33.14	—	—	4:30:03	2.72	3:54:18	408.82
SE-100K	91.52	27:30	42.78	1:22:14	81.67	1:22:00	75.93	—	—	9:40:06	4.82	8:07:29	^*^522.56
SE-150K	91.61	42:30	70.55	2:00:08	130.98	2:00:13	124.27	—	—	—	—	7:47:14	^*^522.63
GB	92.98	01:10	30.02	0:02:45	136.55	0:01:20	48.47	28:55	15.86	0:22:05	9.91	0:34:45	225.57

The read-mapping output is written to /dev/null for this experiment. We also report the mapping rate in percentage (fraction of mapped read over the total number of queried reads). Results are relative to the full-intersection query mode (Algorithm 1). All reported timings are relative to a second run of the experiment, when the index is loaded faster from the disk cache. The “B” query mode of MetaGraph corresponds to the batch mode (with default batch size); and the “NB” corresponds to the non-batch query mode. With a ^*^ we mark the workloads exceeding the available memory ($>500$ GB). For the low-hit workload (a) we use the reads from SRR896663. For the high-hit workload (b) we use the reads from SRR1928200 for *E. Coli*, SRR801268 for *S. Enterica*, and ERR321482 for Gut Bacteria

**Table 6:** Total query time and memory used with Fulgor’s two-pass multi-query optimization compared to “normal” single-pass queries (the same as in Table 5b). Here, we benchmark high-hit workloads using 16 processing threads

	Normal		Two-pass optimization			
	mm:ss	GB	mm:ss	GB	% speedup	%+Mem.
EC	02:10	1.68	01:24	2.78	35.38	65.48
SE-5K	01:16	0.82	01:09	1.47	9.21	79.27
SE-10K	02:26	2.11	02:08	3.18	12.33	50.71
SE-50K	19:15	18.53	11:25	21.12	40.69	13.98
SE-100K	27:30	42.78	23:00	47.16	16.36	10.24
SE-150K	42:30	70.55	35:02	77.47	17.57	9.81
GB	01:10	30.02	01:09	36.47	1.43	21.49

We report the percentage of speedup compared to unoptimized queries as well as the additional memory usage in columns “% speedup” and “%+Mem.”, respectively

**Table 7:** Average index loading time from disk to memory

	Fulgor	Themisto-d1	Themisto-d20	COBS
	mm:ss	mm:ss	mm:ss	mm:ss
EC	00:01	00:02	00:02	00:04
SE-5K	00:01	00:02	00:02	00:05
SE-10K	00:02	00:06	00:06	00:10
SE-50K	00:14	00:40	00:35	03:30
SE-100K	00:30	01:45	01:20	07:15
SE-150K	01:00	02:00	01:00	15:00
GB	00:30	02:00	00:30	00:09

### Comparison of pseudoalignment algorithms on simulated data

To analyze the accuracy of the underlying pseudoalignment algorithms, we perform additional testing with read sets simulated using the Mason [43] simulator. To analyze how mapping and hit rates affect query speed, we simulate a varying proportion of “positive” reads from indexed reference sequences and generate “negative” reads from the human chromosome 19 from the CHM13 v2.0 human genome assembly [44]. We use Fulgor to compare the four mapping algorithms described in "Pseudoalignment algorithms" section.

From Table 8, we see that at various proportions of ground truth positive reads (simulated reads deriving from indexed references), all mapping methods have a true positive rate (TPR), i.e., total reads correctly mapped over the total ground truth positives, greater than 95%. This high sensitivity for all four methods is to be expected since all methods simply check for $k$-mer’s membership to references of origin and do not consider $k$-mer positions in references. One main drawback of eliding positions, heuristically avoiding “locate” queries, and entirely ignoring $k$-mers that are not present in the index, is also clear. All methods incur approximately a 30% false positive rate (FPR), i.e., total reads spuriously mapped over the total ground truth negatives. As is expected, the threshold-union method incurs a slightly higher FPR compared to other methods (30% compared to 27% for other methods) because of its less strict criteria only requiring references to be compatible with $\tau$ fraction of mapped $k$-mers instead of *all* $k$-mers.

**Table 8:** Quality of pseudoalignment algorithms querying 100,000 simulated reads against 50,000 Salmonella genomes indexed with Fulgor

% Positive	Full-intersection		Threshold-union		Kallisto		Alevin-fry	
	TPR	FPR	TPR	FPR	TPR	FPR	TPR	FPR
90	95.0	27.0	97.7	30.0	95.0	27.0	95.1	27.0
70	95.1	27.0	97.7	30.0	95.1	27.0	95.1	27.0
25	95.1	27.0	97.7	30.0	95.2	27.0	95.2	27.0
10	95.5	27.0	97.8	30.0	95.5	27.0	95.5	27.0

We vary the percentage of *positive* reads simulated from indexed Salmonella genomes by diluting queried read sets with *negative* reads simulated from a reference human transcriptome. We consider a mapped positive read (deriving from indexed references) to be a *true positive* if the reference of origin is in the returned set of compatible references; and a mapped negative read (deriving from human chromosome 19) to be a *false positive*. We denote true and false positive rates (%) to be TPR and FPR, respectively. For the threshold-union method, we use $\tau =0.8$

In these benchmarks, we find very little difference in terms of TPR and FPR between the exhaustive methods and skipping heuristics. These results also gesture at one desirable and one undesirable quality of these methods. First, skipping heuristics correctly assume and successfully skip $k$-mers that likely occur on the same unitig and have the same color. Likewise, they have the *potential* to be even more sensitive than the full-intersection method, as they do not, in general, search for every $k$-mer in a query, and can thus avoid scenarios where variation or sequencing errors in a query cause spurious matches to the index, shrinking or eliminating the set of references appearing in the final color assigned to the query. In fact, in a small-scale test, Alanko et al. [15] report that Kallisto’s skipping heuristic results in a small but persistent increase of approximately $0.03\%$ in the mapping rate. However, all four of the pseudoalignment methods evaluated here suffer from a high FPR and low precision. Better algorithms to lower FPR and improve precision without lowering sensitivity too much should be investigated in future work. Such improvements may be possible by adding back information about the *reference positions* where $k$-mers from the query match, incorporating structural constraints [16] or other such restrictions atop the color intersection rule. Yet, those approaches are more computationally involved, require the index to support locate queries, and also substantially diverge from “pseudoalignment ” as traditionally understood. Regardless, we highlight here that Fulgor more easily enables implementing skipping and unitig-based heuristics compared to other methods that do not explicitly store unitig sequences and keep $k$-mers in unitig order. In fact, Fulgor implicitly maintains additional information regarding the *local structural consistency* of $k$-mers. For example, with Fulgor, one can easily check if consecutive $k$-mers are valid on an indexed unitig or check if consecutive unitigs on a read have valid overlaps, in an attempt to reduce the FPR.

## Conclusions and future work

We introduce Fulgor, a fast and compact index for the $k$-mers of a colored compacted de Bruijn graph (ccdBG). Using SSHash, an order-preserving $k$-mer dictionary, Fulgor fully exploits the monochromatic property of unitigs in ccdBGs and implements a very succinct map from unitigs to colors, taking only $1+o(1)$ bits per unitig. Further, Fulgor applies an effective hybrid compression scheme to represent the set of distinct colors.

Across all benchmarked scenarios, Fulgor outperforms Themisto— its most direct competitor—in terms of space *and* speed. Further, though not as small to represent on-disk as MetaGraph’s most space-efficient variant, Fulgor is much faster to query and build, and can be queried with predictable memory usage. In particular, it is worth comparing MetaGraph to other methods. Compared to Themisto and Fulgor that specialize in indexing the colors of a ccdBG, MetaGraph is a *framework* for indexing reference sequences and can be more complicated to build. From our experiments, we argue that Fulgor is the most practical index to use because it is fast to query and its memory usage at query time is frugal and *predictable*—Fulgor does not dynamically decompress metadata at query-time. There is still room for improvement in future work. We discuss some promising directions below.

In terms of speed, we remark that when processing a high-hit workload, the overall runtime is dominated by the time required to *intersect* the colors. As explained in "Exhaustive methods" section, Fulgor currently implements a generic intersection algorithm that only requires two primitive operations, namely next and $\textsc {Next-GEQ}$ (see also "Pseudoalignment algorithms" section). But this is not the only paradigm available for efficient intersection. We could, for example, try approaches that exploit different indexing paradigms, such as Roaring [35] and Slicing [45], that are explicitly designed for fast intersections. These alternative approaches may be significantly faster especially on the high-hit workloads.

Another possible optimization is to implement a caching scheme for frequently occurring and/or recently intersected colors. Caching the uncompressed or intersection-optimized versions of frequently occurring color sets, or previously computed intersections, could speed up query processing substantially when many reads map to the same set of colors.

For example, as we observed, our two-pass query scheme increased query throughput, sometimes considerably, by avoiding completely redundant color intersections. Yet, such an approach, which consists of first collecting color-id lists, then sorting and aggregating identical lists, is simple and only takes advantage of exactly duplicated color-id lists. We note, however, that generalizations of such approaches may be much more powerful and efficient. In general, one can consider ways to take advantage of redundancy and replication in the color-id lists so as to avoid redundant intersections. Specifically, because the intersection operation *distributes* over sub sets, the intersection over a list of color ids can be decomposed into the intersection of the *result* of applying the intersection operation to the sublists that compose the overall list. For example, the intersection $A \cap B \cap C \cap D \cap E = \left( A \cap B \cap C\right) \cap \left( D \cap E\right) = \left( A \cap C\right) \cap \left( B \cap D \cap E\right)$, etc.

This observation leads to the general question of how best to *decompose* the intersection over a multi-set of lists (i.e., the color-id lists taken over all queries) into a collection of redundant sub-problems whose results can be computed once and reused many times. We note similarities to the *frequent itemset mining* problem [46], where one seeks to find subsets of elements that frequently co-occur (in our case, we would be interested in color ids that frequently co-occur in the lists corresponding to queries). However, fully understanding and exploiting the structure of repeated and/or similar patterns in the color lists, and finding “good” factorizations to minimize the computation required to answer the queries, is an interesting and largely unsolved problem in its own regard. We leave the question of how best to approach this problem, and, in general, how to optimize different pseudoalignment algorithms for multiple query patterns rather than considering each query independently, to future work.

In terms of space, one property that Fulgor does not exploit in this work is the fact that *many* unitigs in the ccdBG share *similar colors*—i.e., co-occur in many reference sequences. This is so because unitigs arising from conserved genomic sequences will share similar occurrence patterns. We point out that we have recently proposed a method to take advantage of this redundancy in a related line of research [47]. Other works have also explored this possibility. For example, Almoradesi et al. [48] developed a method that efficiently compresses distinct, but highly-correlated colors, through a variant of referential encoding. Specifically, they compute a minimum spanning tree (MST) on a sub-graph of the color graph induced by the ccdBG, and encode a color by recording its differences with respect to its parent in the MST. This vastly reduces the space required to encode the color set when many similar colors exist, as we would expect in a pangenome, and fast query speed can be retained through color caching. Another related approach would be to resort to clustering similar colors and encoding all colors within a cluster with respect to a cluster representative color [49]. Likewise, although not specifically designed to compress colors, MetaGraph and its variants can exploit similarity between colors using a general compression scheme that records differences in stored metadata (in this case, the colors) between adjacent $k$-mers [21]. We lastly note that, since the colored $k$-mer indexing problem is *modular* ("Modular indexing layout" section), novel relational compression techniques for the set of distinct colors can be developed and optimized independently of the other components of the index.

Finally, in our experiments with simulated data analyzing the quality of pseudoalignment algorithms from "Comparison of pseudoalignment algorithms on simulated data" section, we find higher than desirable false positive rates. This suggests that, at least for the metagenomic and pangenomic reference collections where many references share similar $k$-mer content, better read-mapping heuristics and algorithms that improve specificity (i.e., reduce the spurious mapping of reads not arising from indexed references) without trading-off too much recall are still sorely needed. Here, it will be desirable to search for methods that can improve specificity without the need to retain reference positions or issue locate queries for all $k$-mers. We suggest that there may be several promising directions. For example, one may consider enforcing local structural consistency among matched $k$-mers to potentially reduce spurious mapping. Likewise, one may consider filtering repetitive and low-complexity $k$-mers from contributing to the final pseudoalignment result. Finally, by analogy to BLAST [50], one may consider evaluating the likelihood that a pseudoalignment result is spurious by comparing the matching rate against against some null or background expectation to account for the fact that, in very large reference databases, a very small number of (potentially correlated) $k$-mers may be insufficient evidence to consider a query as compatible with a subset of references.

## Data Availability

**Software** The Fulgor software is available on GitHub at https://github.com/jermp/fulgor. Scripts to reproduce the experiments in the article are instead available at https://github.com/jermp/fulgor-benchmarks. **Datasets** The Salmonella enterica genomes can be downloaded from http://ftp.ebi.ac.uk/pub/databases/ENA2018-bacteria-661k. The Escherichia coli genomes are available on Zenodo at https://zenodo.org/record/6577997. The 30,691 bacteria genomes from the human gut can be downloaded from https://arken.nmbu.no/~larssn/humgut/index.htm.

## Footnotes

1 Commit hash 0d98923 (23/12/2023): github.com/jermp/fulgor/kallisto_psa/psa.cpp.

2 Commit hash 0d98923 (23/12/2023): github.com/jermp/fulgor/piscem_psa/hit_searcher.cpp.

3 https://www.ebi.ac.uk/ena/browser/view/SRR1928200.

4 https://www.ebi.ac.uk/ena/browser/view/SRR896663.

5 https://www.ebi.ac.uk/ena/browser/view/SRR801268.

6 https://www.ebi.ac.uk/ena/browser/view/ERR321482.
